# Macrophage–Fibroblast Crosstalk Governs Tumor Mechanics, as Revealed by Spatiotemporal Force Mapping in 3D Spheroids

**DOI:** 10.1002/adhm.71844

**Published:** 2026-10-16

**Authors:** Kunal P. Pednekar, Daan Vorselen, Jai Prakash

**Affiliations:** ^1^ Department of Medical Biosciences Radboud University Medical Centre Nijmegen the Netherlands; ^2^ Department of Bioengineering Technologies University of Twente Enschede the Netherlands; ^3^ Department of Cell Biology & Immunology (CBI) Wageningen University & Research Wageningen the Netherlands; ^4^ Center for Translational Immunology University Medical Center Utrecht and Utrecht University Utrecht the Netherlands

**Keywords:** 3D cancer models, extracellular matrix, fibroblasts, force mapping, macrophages, tumor spheroids, tumor stroma

## Abstract

Understanding how stromal and immune cells regulate tumor mechanics is essential for deciphering cancer progression and therapy resistance. This study presents a microparticle‐based traction force microscopy strategy for in situ quantification of compressive forces within 3D tumor spheroids. Multicellular spheroids composed of tumor cells (T), fibroblasts (F), and macrophages (M) were generated to mimic the tumor microenvironment and to understand the contribution of fibroblast–macrophage crosstalk in tumor mechanobiology. Tri‐culture spheroids exhibited the highest proliferative capacity compared to mono‐ and bi‐cultured tumor spheroids. Gene expression profiling indicated strong fibroblast activation, extracellular matrix remodeling, and M2‐like macrophage polarization, highlighting a pro‐tumoral phenotype. Incorporation of deformable hydrogel microparticles allowed direct mapping of local compressive and indirect quantification of anisotropic stresses. This mapping revealed fibroblasts are the principal source of force generation with the spheroids. Notably, macrophages amplified bulk and anisotropic stresses over two‐fold through paracrine activation of fibroblasts. Spatial mapping showed pressure gradients from the core to the periphery, while temporal analyses demonstrated a progressive increase in mechanical stresses with spheroid growth. Together, these findings reveal a surprising governing of intratumoral mechanics by macrophage–fibroblast interactions and demonstrate the potential of microparticle‐based 3D force mapping within complex tumor environments.

## Graphical abstract

This study shows crosstalk among tumor cells, fibroblasts, and macrophages alters the physical forces inside 3D tumor spheroids. Incorporating deformable microparticles in 3D tumor spheroids, the researchers mapped mechanical stresses spatially and with respect to tumor growth (temporally). They showed that fibroblasts are mainly involved in stress generation, which is further amplified with their crosstalk with the macrophages. Schematics were created by Biorenders.com.

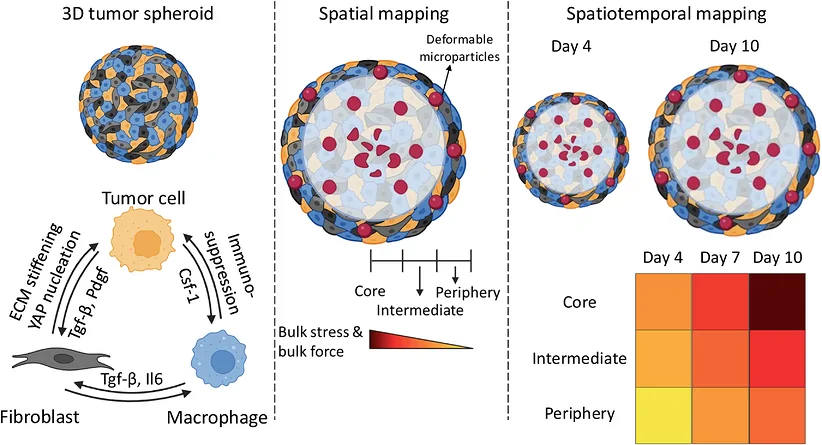

## 1 Introduction

Cells constantly generate and sense mechanical forces within their local microenvironments, enabling them to probe the extracellular landscape and respond to biochemical and biophysical cues [1, 2]. Such mechanical interactions not only control fundamental cellular processes such as migration, proliferation, and differentiation, but also play a pivotal role in shaping tissue architecture and disease progression [3]. In solid tumors, the dense desmoplastic stroma, driven largely by excessive deposition and remodeling of the extracellular matrix (ECM), gives rise to elevated solid stress and tissue stiffness. These are key mechanical features that promote tumor progression, metastasis, and therapeutic resistance [4]. Increased stiffness has been shown to enhance malignant cell proliferation, migration, and invasive potential due to activation of mechanotransduction pathways such as YAP/TAZ and integrin‐mediated signaling [5, 6, 7, 8].

Among different stromal components, cancer‐associated fibroblasts (CAFs) are key drivers of desmoplasia. CAFs are reciprocally activated by paracrine signals from tumor cells, including TGF‐β, IL‐1β, and PDGF, and in turn secrete large amounts of fibrillar ECM proteins, including collagens and fibronectin, and remodel them and reinforce the tissue stiffness [9, 10]. This reciprocal activation between tumor cells and CAFs establishes a feed‐forward loop that sustains tumor growth and promotes an aggressive phenotype [11].

Beyond CAFs, emerging evidence indicates that immune cell components, particularly macrophages, also control the tumor mechanical properties [12, 13, 14]. Tumor‐associated macrophages (TAMs) can directly modulate fibroblast activation as well as ECM remodeling through cytokine signaling (e.g., TGF‐β) and matrix‐degrading enzymes such as MMPs and ADAMs [14, 15]. Macrophage–fibroblast crosstalk has been shown to regulate collagen alignment and tissue stiffness in tumors and fibrotic lesions alike [15, 16]. However, the precise mechanisms through which macrophages contribute to the biophysical landscape of the TME remain poorly understood.

Studying these dynamic cell–cell interactions and their mechanical consequences in in vivo models is challenging. In murine tumor models, spatial heterogeneity, limited imaging resolution, and the complex interplay among multiple stromal and immune cell types hinder quantitative assessment of mechanical forces. Therefore, simplified yet physiologically relevant three‐dimensional (3D) tumor models are instrumental for dissecting the cellular and mechanical dynamics under rather controlled conditions [17, 18, 19].

While macroscopic methods such as atomic force microscopy (AFM) and rheology have provided global measures of tumor stiffness, these approaches do not quantify the intratumoral solid stresses. Moreover, these methods lack the spatial and temporal resolution required to capture heterogeneity in force distribution within tumors [20, 21]. Traction force microscopy (TFM) presents a powerful method to study cellular force generation and mechanotransduction with high precision. However, conventional TFM is largely confined to 2D systems, limiting its relevance for complex 3D interactions and multicellular assemblies [22, 23]. Recent advances in force sensing technology use hydrogel‐based microparticles, with tunable stiffness mimicking cellular elasticity to quantitate bulk, normal, and shear stresses in 3D environments [24, 25, 26, 27] and during ligand‐dependent interactions such as phagocytosis or immune synapse formation [22, 24]. Despite these advances, spatial analysis of both bulk and anisotropic stresses in tumors and applications to complex multicellular tumor models remain limited [22, 24].

In this study, we utilize an established microparticle‐based force sensing strategy to quantify mechanical forces within 3D microenvironments in a spatiotemporal manner. We aimed to first determine whether and how macrophages modulate the generation of mechanical stresses within tumors. Subsequently, we aimed to explore how macrophage–fibroblast crosstalk modulates the tumor growth, phenotypic alterations, and intratumoral mechanical forces in a spatial (core to periphery) and time‐dependent manner as a simple yet physiologically relevant model system. To address these questions, we developed heterotypic multicellular spheroids composed of tumor cells alone, with fibroblasts, with macrophages, or together with both fibroblasts and macrophages. We characterized these spheroids in terms of proliferation and gene expression related to activation and phenotype. To directly quantify mechanical forces, deformable microparticles of defined stiffness were incorporated into the spheroids generated from colorectal cancer (murine model) and pancreatic ductal adenocarcinoma (PDAC, human model), providing a comprehensive readout of local forces. Using confocal microscopy and 3D reconstruction tools, the compressive and anisotropic forces locally exerted within the spheroids were quantified. This strategy enabled us to generate high‐resolution maps of mechanical forces within spheroids, capturing both spatial gradients and temporal dynamics of tumor solid stresses.

## 2 Results

### 2.1 Tumor Cell–Fibroblast–Macrophage Interaction Stimulates Cell Proliferation in 3D Culture

We established a 3D colon carcinoma model comprising CT26 tumor cells, NIH‐3T3 fibroblasts, and RAW264.7 macrophages, as well as a 3D PDAC model comprising PANC‐1 tumor cells, pancreatic stellate cells (PSCs), and THP‐1 human monocytes. Four spheroid types were generated: tumor‐only (T), tumor + fibroblasts (T + F), tumor + macrophages (T + M), and tumor + fibroblasts + macrophages (T + F + M) (Figure 1a). Brightfield imaging at Days 3, 5, and 7 for both 3D spheroid models showed distinct growth patterns among the groups (Figure 1b).

**FIGURE 1:**
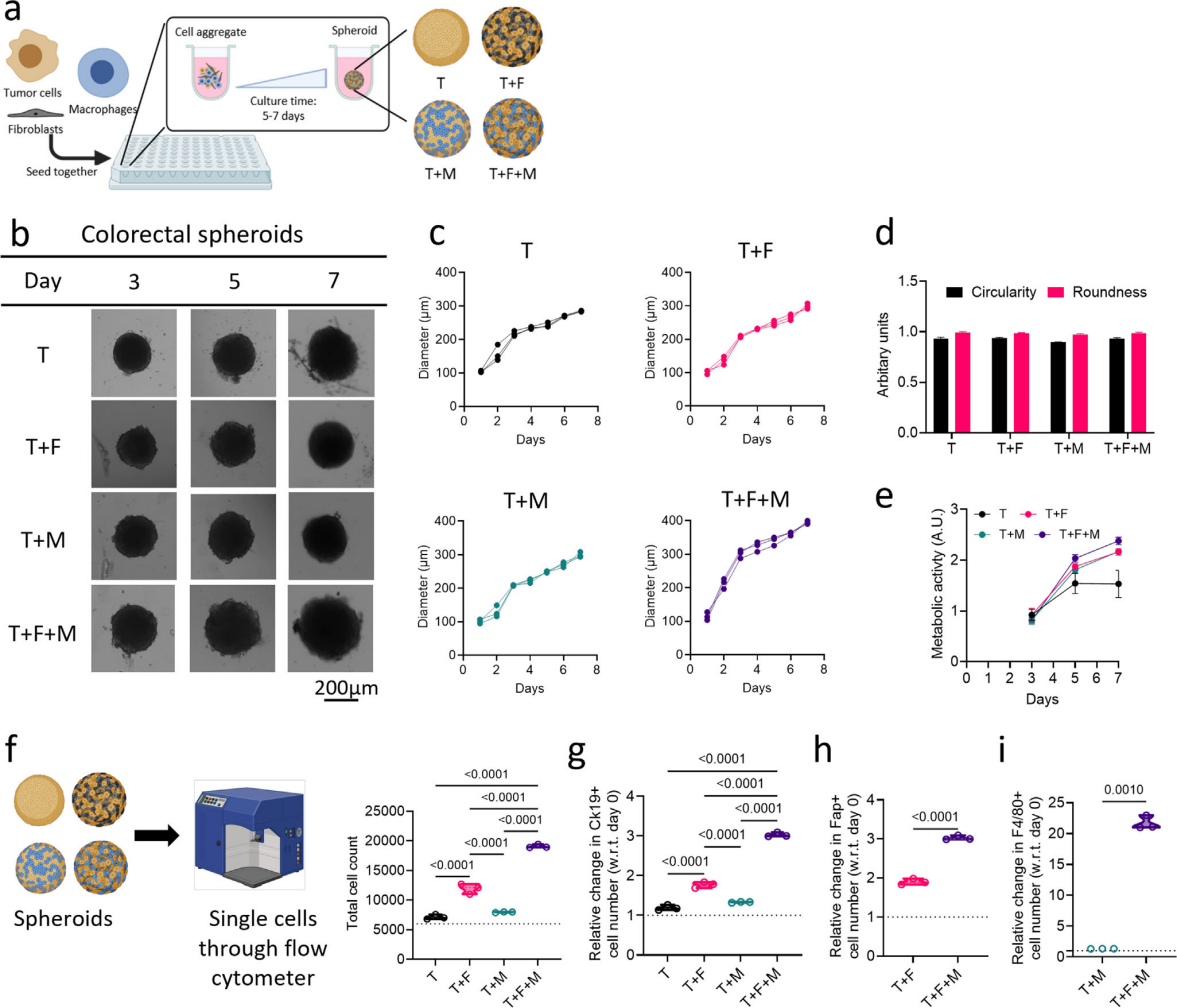
Spheroid growth and proliferation in mono‐ and co‐culture spheroids. (a) Schematic representation of spheroid formation using tumor cells, fibroblasts, and macrophages either alone or in co‐culture with others, (b) Representative brightfield microscopic images of colorectal spheroids formed using the tumor cells alone, or either co‐cultured with fibroblasts or macrophages, or both fibroblast, and macrophages, (c) Line graph showing the growth curve of colorectal spheroids as measured from the brightfield microscopic images. Each point is an average of at least 10 spheroids (technical replicates), and each line is a biological replicate of the spheroid growth, (d) Bar graph showing the circularity and roundness of different types of colorectal spheroids, (e) Line graph showing the metabolic activity of the colorectal spheroids at different timepoints. Each point is the average of 3 biological replicates, (f) Schematic representation of different colorectal spheroids collected for flow cytometric analysis and bar graph showing the total cell count in different spheroids 7 days post seeding, dotted line represents the initial seeding density of the spheroids, (g) Bar graph showing the relative change in the count of tumor cells (Ck19+ cells) in different types of spheroids, (h) Bar graph showing the relative change in the count of fibroblasts (Fap+ cells) in different types of spheroids, (i) Bar graph showing the relative change in the count of macrophages (F4/80+ cells) in different types of spheroids. Data represented as mean ± standard error of mean (SEM). Statistical analysis for multiple groups was performed with one‐way ANOVA (Tukey), while comparing two groups, unpaired two‐tailed Student's *t*‐test was used. Schematics created using Biorender.com.

For the colorectal spheroids, tumor‐only and T + M spheroids reached diameters of ∼280 µm by Day 7, whereas fibroblast‐containing spheroids grew to ∼300 µm in T + F and ∼400 µm in T + F + M (Figure 1b,c). Quantification of circularity and roundness indicated that all groups formed compact, uniformly shaped spheroids (Figure 1d). Metabolic activity, measured by CellTiter‐Glo assay, increased over time in all spheroid types (Figure 1e).

For the PDAC spheroids, tumor‐only spheroids reached diameters of ∼290 µm by Day 7, whereas fibroblast‐containing spheroids grew to ∼305 µm in T + F and ∼420 µm in T + F + M (Figure S1a,b). However, the T + M condition did not form spheroids because of the outgrowth of the monocytes/ macrophages on Day 5 dismantling the spheroid formation. To ensure the growth differences were not influenced by core necrosis, we performed Live/Dead staining. We observed high viability (> 95%) across all conditions (Figure S1c).

Flow cytometry of dissociated Day 7 colorectal spheroids revealed differences in overall cell proliferation (Figure 1f). Despite initial seeding of 6000 cells across all groups, tumor‐only spheroids showed a modest 1.25‐fold increase relative to initial seeding, while T + M spheroids proliferated ∼1.4‐fold. Co‐culture with fibroblasts led to a 2‐fold increase in T + F spheroids, and the presence of both fibroblasts and macrophages resulted in a nearly 3‐fold increase in T + F + M spheroids (∼18 000 cells, Figure 1f).

Analysis of individual cell populations showed interesting outcomes. Relative to their initial seeding density, tumor cells (Ck19+ cells) in tumor‐only spheroids and T + M spheroids showed a modest increase of 1.2 to 1.3‐fold. While tumor cells in T + F and T + F + M spheroids showed a relative increase in their number of 1.8‐fold and 3‐fold respectively (Figure 1g). Fibroblast (Fap+ cells) relative numbers were also enhanced in the presence of both tumor cells and macrophages (Figure 1h). Macrophages exhibited the highest relative expansion, showing up to a 20‐fold increase in T + F + M spheroids (Figure 1i). These results indicate that fibroblast–macrophage interactions markedly enhance growth of all constituent cell types.

### 2.2 Tumor Cell–Fibroblast–Macrophage Interaction Drives Pro‐Tumoral Phenotypes

Gene expression profiling revealed coordinated changes in epithelial, stromal, ECM, and immune‐related markers across spheroid types (Figure 2a). E‐cadherin levels progressively decreased from tumor‐only spheroids to T + F + M spheroids (Figure 2b). The bulk gene expression reflects the total cellular composition. To avoid the additive effect, the gene expression for cell specific genes was normalized with the number of cells present in the spheroid on Day 7. In case of E‐cadherin, except for T + F spheroids, other spheroid types showed no change in the fold change (Figure 2c). For another tumor cell specific marker Ck19, we observed a 3‐fold and 6‐fold upregulation in the T + F spheroids and T + F + M spheroids respectively (Figure 2b). This trend was also true when normalized to the tumor cell count, with the T + F and T + F + M spheroids showing approximately 12–13‐fold upregulation (Figure 2c).

**FIGURE 2:**
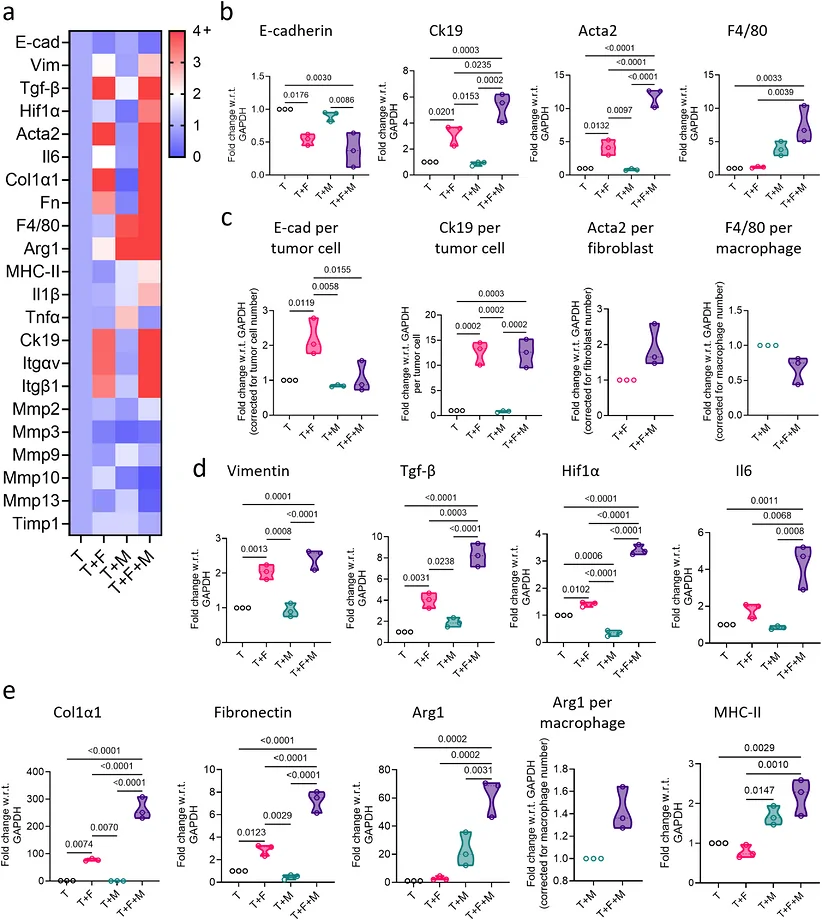
Gene expression of different types of spheroids. (a) Heatmap showing the gene expression changes in the spheroids with monoculture and co‐cultured either with fibroblast alone, or macrophages alone, or together with fibroblast and macrophages. (b) Bar graph showing the comparative gene expression for E‐cadherin, Ck19, Acta2 (or α‐smooth muscle actin, α‐Sma, and F4/80, (c) Bar graph showing the fold change of E‐cadherin, Ck19, Acta2, and F4/80 normalized for the specific cell type present in different types of colorectal spheroids on Day7, (d, e) Bar graph showing the comparative gene expression for Vimentin, Tgf‐β, Hif1α, Il6, Col1α1, Fibronectin, Arginase1, and MHC‐II. The expression levels in mono‐spheroids were set at 1.0 and other groups were compared to them. Data represented as mean ± standard error of mean (SEM). Statistical analysis for multiple groups was performed with one‐way ANOVA (Tukey) and for comparing two groups unpaired student's *t*‐test was performed.

Fibroblast activation markers followed similar trends. Acta2 expression increased 5‐fold in T + F spheroids and ∼12‐fold in T + F + M spheroids (Figure 2b). Acta2 when normalized with the number of fibroblasts, we observed a 2‐fold upregulation for the T + F + M spheroids as compared to the T + F spheroids (Figure 2c). Similarly, F4/80 a macrophage specific gene was upregulated ∼4‐fold in the T + M spheroids while 6‐fold in the T + F + M spheroids (Figure 2b). Interestingly, the gene expression when normalized with the number of macrophages, we observed a 0.8‐fold downregulation in T + F +M spheroids as compared to the T + M spheroids (Figure 2c).

Vimentin expression was unchanged in T and T + M spheroids but increased more than 2‐fold in fibroblast‐containing spheroids (Figure 2d). Tgf‐β and Hif1α were markedly increased in T + F + M spheroids, showing ∼8‐fold and ∼3‐fold upregulation respectively (Figure 2d). Similarly, Il6 expression increased modestly in T + F spheroids and further to ∼5‐fold in T + F + M spheroids (Figure 2d).

ECM‐related genes were strongly upregulated in fibroblast‐containing spheroids. Collagen (Col1α1) increased ∼200‐fold in T + F spheroids and ∼300‐fold in T + F + M spheroids (Figure 2e). Fibronectin also showed an ∼8‐fold increase in T + F + M spheroids (Figure 2e). Integrins Itgαv and Itgβ1 were elevated 5–10‐fold in T + F and T + F + M spheroids (Figure S2).

Macrophage markers confirmed increased macrophage numbers and M2‐like polarization. Arginase1 increased 20‐fold in T + M and ∼60‐fold in T + F + M spheroids, whereas MHC‐II showed only modest induction (Figure 2e). Interestingly, Arginase 1 when normalized with the number of macrophages, we observed a 1.4‐fold upregulation in the T + F + M spheroids compared to the T + M spheroids (Figure 2e). Il1β increased moderately in T + M and T + F + M spheroids, while Tnfα increased in T + M but declined in T + F + M spheroids (Figure S2).

Matrix metalloproteinases showed context‐dependent changes. Mmp‐2 was modestly upregulated in T + F + M spheroids (Figure S2), while Mmp‐3, Mmp‐10, and Mmp‐13 were downregulated across spheroid types (Figure S2). Mmp‐9 was elevated in T + M but not in fibroblast‐containing spheroids (Figure S2). Timp‐1 exhibited no consistent pattern (Figure S2).

Overall, these findings show that combined tumor–fibroblast–macrophage interactions synergistically enhance epithelial plasticity, fibroblast activation, ECM deposition, and M2‐like macrophage polarization, collectively driving a strongly pro‐tumoral molecular phenotype.

### 2.3 Fibroblast‐Macrophage Crosstalk Induces Yap‐Dependent Mechanosensing

To understand the crosstalk between the three cell types, immunofluorescence staining was performed for Ck19, αSMA, Col1, F4/80, Arg1, and Yap. To understand the spatial localization of the cells in the multicellular spheroids, the spheroids were first divided into three spatial regions: the core (0–75 µm radius), the intermediate zone (75–150 µm), and the periphery (> 150 µm) (Figure S3a). Analyzing the sections, revealed the compactness of the spheroids. The tumor‐only, T + F, and T + F + M spheroids were tightly packed while the T + M spheroids were loosely packed and porous (Figure S3b).

Ck19, an epithelial marker, showed overall decrease in the expression in the fibroblast containing spheroids. In tumor‐only, T + F and T + F + M spheroids, the Ck19 was spatially localized homogenously over the spheroid, while for the T + M the staining was more localized in the intermediate region and slightly lower in the core and periphery (Figure 3).

**FIGURE 3:**
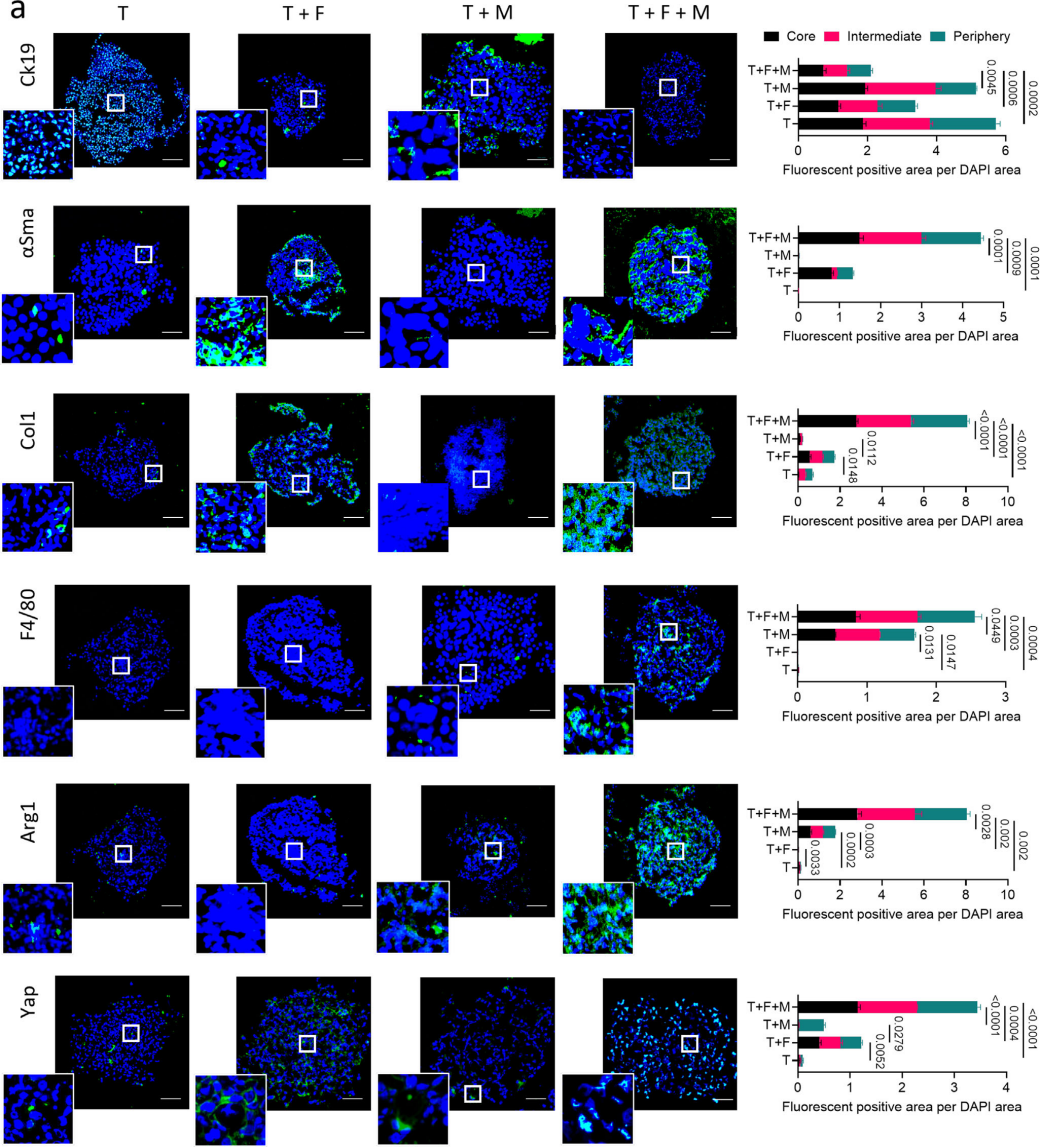
Macrophage‐fibroblast crosstalk induces pro‐tumoral protein expression in tri‐culture spheroids. Representative immunofluorescent images of different types of spheroids showing the staining (pseudocolor green, pseudocolor blue for nuclei) and their spatial quantification for Ck19, αSma, collagen1, F4/80, Arg1, and Yap. Insets shown in white box show a zoom‐in region of interest. Scalebar for all the staining is 100 µm. Data represented as mean ± standard error of mean (SEM). Statistical analysis for multiple groups was performed with one‐way ANOVA (Tukey).

αSMA staining a prominent marker for activated fibroblast was observed in the T + F and T + F + M spheroids. The fibroblasts were localized in the core and the periphery for T + F spheroids, while the spatial localization of fibroblasts in T + F + M spheroids was more homogenous. The activation of fibroblast occurs when in contact with the tumor cells which was even prominent with the presence of macrophages along with the tumor cells (Figure 3). Fibroblast activation in general leads to the deposition of collagen. Collagen1 staining showed a similar trend to that of activated fibroblasts, with the T + F spheroids showing higher signal of collagen which was enhanced strongly in the presence of macrophages in the T + F + M spheroids (Figure 3). Interestingly, low collagen staining was also observed in the tumor‐only and T + M spheroids. Collagen1 was homogenously distributed in the T + F + M spheroids.

To localize the macrophages, F4/80, a pan‐macrophage marker was used. The macrophages were randomly localized in both T + M and T + F + M spheroids suggesting functional interaction with the tumor cells and the fibroblasts (Figure 3). Prominent and homogenous Arginase1 staining in the T + F + M spheroids showed the presence of M2‐like macrophages in the T + F + M spheroids (Figure 3). Lastly, Yap staining provided insights into mechanotransduction activity within the spheroids. Tumor‐only and T + M spheroids displayed weak cytoplasmic Yap localization, indicating low mechanical tension. In T + F spheroids, Yap signal increased and partially localized to the nuclei, suggesting activation of Yap‐dependent mechano‐signaling due to enhanced stromal contractility. Interestingly, the Yap staining localized in the nuclei was observed higher in the core region of the spheroid. In T + F + M spheroids, Yap staining was markedly elevated and predominantly nuclear, consistent with the highest levels of mechanical stress (Figure 3). Moreover, the nuclear localization of Yap staining was homogenous with some cells in the intermediate and periphery showing inactive intracellular Yap signal.

Together, these analyses reveal that fibroblasts and macrophages together lead to enhanced fibroblast activation, collagen deposition, macrophage polarization, and Yap‐driven mechanotransduction.

### 2.4 Incorporation of Deformable Microparticles in Spheroids

To quantify local mechanical forces within multicellular spheroids and to study the effect of fibroblasts and macrophages on these mechanical forces, we incorporated deformable BSA‐coated fluorescent microparticles during spheroid self‐assembly (Figure 4a). Confocal z‐stacks confirmed successful incorporation in all spheroid types (Figure 4b,c). In both colorectal and PDAC spheroids, tumor‐only, T + F, and T + F + M spheroids contained similar numbers of microparticles (40–50 per *z*‐stack), whereas T + M colorectal spheroids incorporated fewer microparticles (Figure 4d). The distribution of microparticles, when analyzed spatially in the defined regions was largely similar for the different spheroid types (Figure 4e).

**FIGURE 4:**
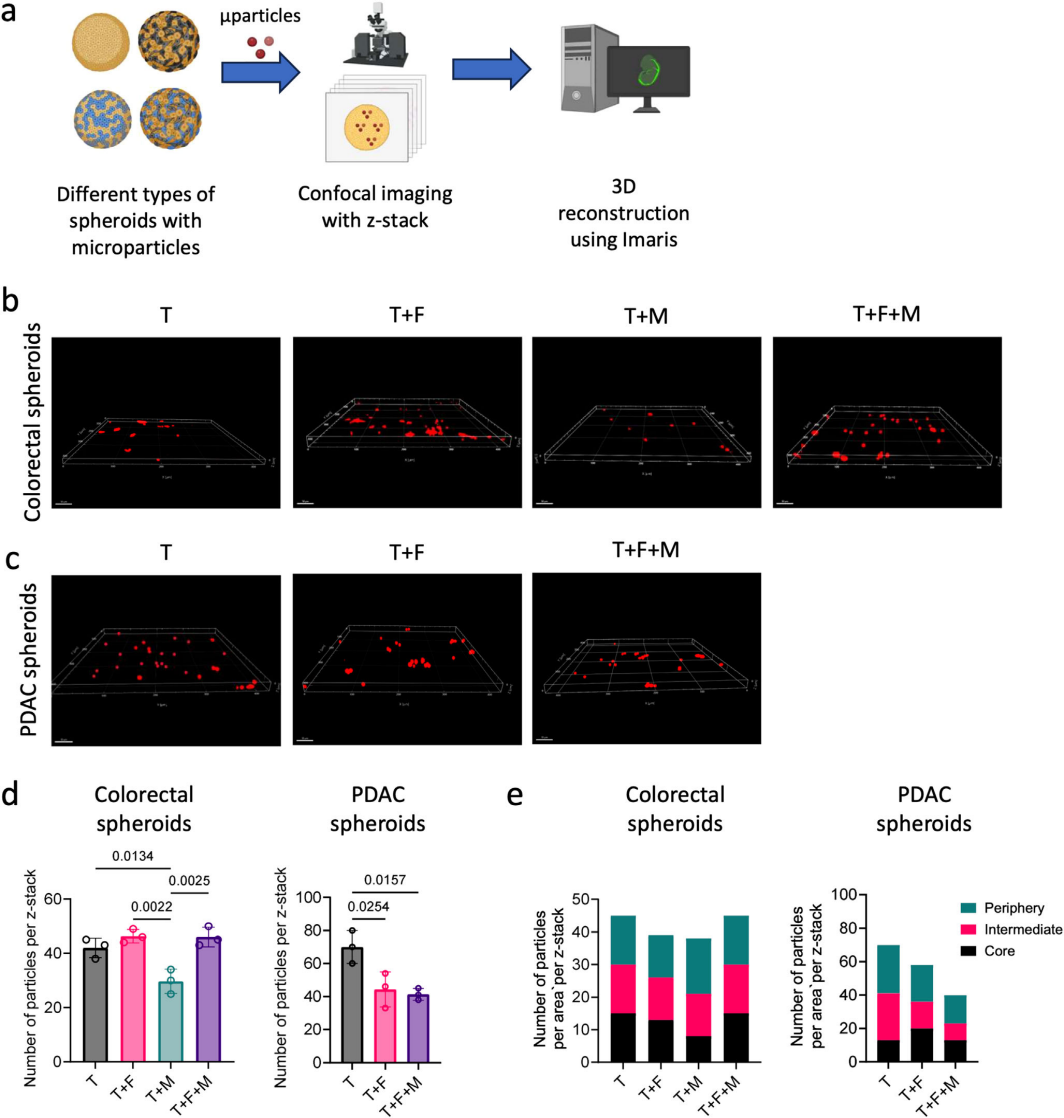
Microparticle incorporation and spatial localization in spheroids. (a) Schematic representation of different types of spheroids incorporating microparticles during self‐assembly and spheroid formation which are analyzed using confocal microscopy and 3D reconstruction using IMARIS software, (b, c) Representative microscopic images of z‐stacks representing the distribution of microparticles (in red) inside different types of colorectal (b) and PDAC (c) spheroids, (d) Bar graph representing the distribution of microparticles in different types of colorectal and PDAC spheroids per z‐stack, (e) Bar graph representing the microparticles incorporated in different regions for various types of colorectal and PDAC spheroids per z‐stack. Data represented as mean ± standard error of mean (SEM). Statistical analysis for multiple groups was performed with one‐way ANOVA (Tukey). Schematics created using Biorender.com.

Microparticle incorporation and spatial mapping indicate that while macrophages reduce particle uptake, the presence of fibroblasts and macrophages does not markedly alter the radial distribution of embedded particles within spheroids.

### 2.5 Spatial Mapping of Microparticle Deformation Reveals Fibroblast‐ and Macrophage‐Driven Forces Within Spheroids

Quantitative analyses of microparticle geometry from confocal microscopy z‐stacks in colorectal and PDAC spheroids were performed using IMARIS. Prior to analysis, a threshold was set to allow for Gaussian surface smoothening. The position, sphericity, and deformed volume of individual microparticles was then analyzed. Significant differences in microparticle deformation were observed across spheroid types (Figure 5a, Movie S1).

**FIGURE 5:**
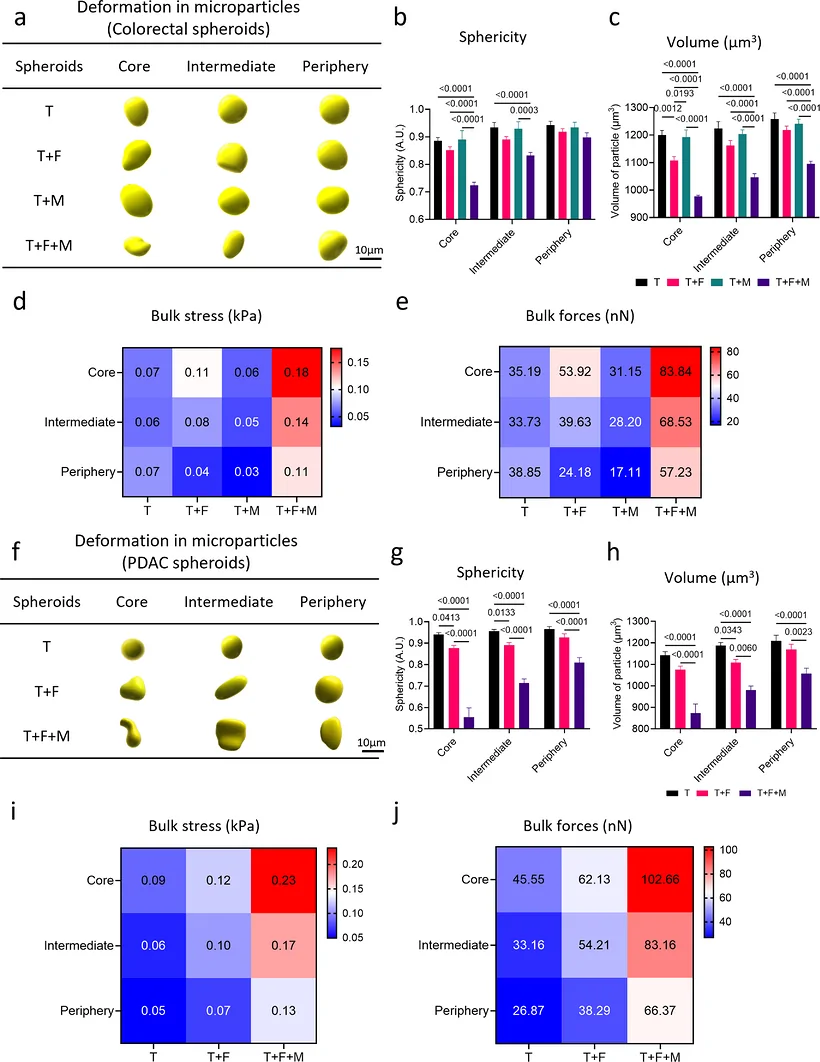
Spatial mapping of microparticle deformation reveals fibroblast‐ and macrophage‐driven bulk compressive stresses. (a) Representative images of deformed microparticles in different regions of different kinds of colorectal spheroids showing the change in sphericity due to the mechanical forces applied on them post spheroid formation; (b) Quantification of the sphericity of the microparticles present in different regions in different kinds of colorectal spheroids; (c) Quantification of the sphericity of the microparticles present in different regions in different kinds of colorectal spheroids; (d) Heatmap showing the quantification of bulk compressive stress in different regions of tumor only, T + F, T + M, and T + F + M colorectal spheroids as calculated based on the volumetric strain on the microparticles; (e) Heatmap showing the quantification of bulk compressive forces acting on the microparticles in different regions of tumor only, T + F, T + M, and T + F + M colorectal spheroids as calculated based on the pressure exerted on the microparticles, (f) Representative images of deformed microparticles in different regions of different kinds of PDAC spheroids showing the change in sphericity due to the mechanical forces applied on them post spheroid formation; (g) Quantification of the sphericity of the microparticles present in different regions in different kinds of PDAC spheroids; (h) Quantification of the sphericity of the microparticles present in different regions in different kinds of PDAC spheroids; (i) Heatmap showing the quantification of bulk compressive stress in different regions of tumor only, T + F, T + M, and T + F + M PDAC spheroids as calculated based on the volumetric strain on the microparticles; (j) Heatmap showing the quantification of bulk compressive forces acting on the microparticles in different regions of tumor only, T + F, T + M, and T + F + M PDAC spheroids as calculated based on the pressure exerted on the microparticles. Data represented as mean ± standard error of mean (SEM). Statistical analysis for multiple groups comparison was performed with two‐way ANOVA (Tukey).

Quantitative analysis (mentioned in text as mean ± SEM) of sphericity and volume of these deformed microparticles confirmed the visual observations (Figure 5b,c). In the core, sphericity dropped to 0.72 ± 0.011 in T + F + M spheroids compared with 0.85 ± 0.012 in T + F spheroids and 0.89 ± 0.012 in tumor‐only and T + M spheroids. Similar trends were observed in the intermediate zone (0.83 ± 0.011 in T + F + M ‘s, 0.89–0.93 for other spheroids) and in the periphery (0.89 ± 0.017 in T + F + M s compared with 0.92–0.95 in the other spheroids) (Figure 5b). While undeformed microparticles had an initial volume of approximately 1300 µm^3^, volumes of microparticles in the core region of T + F + M spheroids dropped to about 977 ± 4 µm^3^, significantly lower than T + F (1107 ± 14 µm^3^) or tumor and T + M cores (1175–1210 µm^3^) (Figure 5c). Intermediate and peripheral regions followed the same trend, with the greatest reduction in volume being consistently observed in T + F + M spheroids.

Furthermore, we quantified the bulk stresses and bulk forces acting on the deformable microparticles based on their volume changes. In tumor spheroids without macrophages and fibroblasts, bulk stresses were relatively low and exhibited only slight spatial variation, ranging from 0.06 to 0.075 kPa in all regions (Figure 5d, Figure S4a). The corresponding bulk forces on a cell‐sized object ranged between 35 ± 11 and 38 ± 16 nN in the periphery) (Figure 5e, Figure S4b), consistent with the limited microparticle deformation observed in the microparticles. T + F spheroids exhibited core stress of 0.105 ± 0.028 kPa, intermediate stress of 0.072–0.08 kPa, and peripheral stress of 0.042–0.047 kPa (Figure 5d, Figure S4c). This translated into bulk force values of 54 ± 13 nN in the core, decreasing to 24 ± 15 nN at the periphery (Figure 5e, Figure S4d). T + M spheroids showed a similar mechanical profile to tumor spheroids, with stress of 0.06 ± 0.04 kPa in the core and intermediate regions that decreased toward the periphery to about 0.032 ± 0.04 kPa (Figure 5d, Figure S4e). The derived forces followed the same trend, ranging from 31 ± 20 nN in the core to 17 ± 20 nN at the periphery, further supporting the observation that macrophages alone do not significantly increase spheroid contractility (Figure 5e, Figure S4f).

Notably, the highest bulk stresses were detected in spheroids containing both fibroblasts and macrophages. In these spheroids, stress reached 0.175 ± 0.01 kPa in the core, with intermediate and peripheral values of 0.14 ± 0.03 and 0.11 ± 0.02 kPa, respectively (Figure 5d, Figure S4g). The corresponding forces peaked at 85 ± 3 nN in the core, followed by 69 ± 11 nN in the intermediate region, and 57 ± 9 nN even at the periphery (Figure 5e, Figure S4h).

PDAC spheroids showed an even pronounced effect on the microparticle deformation in spatial analysis (Figure 5f, Movie S2). IMARIS analysis confirmed the deformations in the microparticles spatially and quantitatively. Sphericity in the core was approximately 0.94 ± 0.01 and 0.88 ± 0.013 for tumor‐only and T + F spheroids. Interestingly, the core of the T + F + M spheroids showed a strong deformation of the microparticles with the sphericity being 0.55–0.6. Similar trends were observed in the intermediate region where the sphericity dropped from 0.95 ± 0.01 in tumor‐only spheroids to 0.71 ± 0.02 in T + F + M spheroids while for the periphery it ranged from 0.96 ± 0.01 in tumor‐only to 0.92 ± 0.017 in T + F and 0.8 ± 0.022 in T + F + M spheroids (Figure 5g).

The volumes of the microparticles incorporated in the PDAC spheroids also showed a significant compression (Figure 5h). For the T + F spheroids, the volume of microparticles in the core regions dropped down to 1075 ± 16 µm^3^ and the volumes further dropped in the T + F + M spheroids to a value of 875 ± 42 µm^3^. In case of intermediate (1187 ± 14 µm^3^) and periphery (1210 ± 25 µm^3^) regions, the volume of microparticles in the tumor‐only spheroids were slightly higher than the core. Similarly, for the T + F spheroids, the volume decreased from 1168 ± 24 µm^3^ in the periphery, 1100 ± 14 µm^3^ in the intermediate regions, and 1075 ± 16 µm^3^ in the core of the spheroids. The T + F + M spheroids again showed a higher compression with the volume in the periphery being 1050 ± 24 µm^3^ and the volumes in the core being 872 ± 42 µm^3^.

Next, we quantified the bulk stress and bulk force acting on the deformed microparticles in the PDAC spheroids. In tumor‐only spheroids, bulk stresses were relatively low and exhibited only slight spatial variation, ranging from 0.05 to 0.09 kPa in all regions (Figure 5i, Figure S4i). The corresponding bulk forces on the microparticles ranged between 26–45 nN (Figure 5j, Figure S4j), consistent with the limited microparticle deformation observed in the microparticles. T + F spheroids exhibited core stress of 0.12 ± 0.01 kPa, intermediate stress of 0.1 ± 0.01 kPa, and peripheral stress of 0.07 ± 0.013 kPa (Figure 5i, Figure S4k). This translated into bulk force values of 62 ± 4 nN in the core, decreasing to 38 ± 6 nN at the periphery (Figure 5j, Figure S4l).

Notably, the highest bulk stresses were detected in spheroids containing both fibroblasts and macrophages. In these spheroids, stress reached 0.23 ± 0.02 kPa in the core, with intermediate and peripheral values of 0.17 ± 0.01 and 0.13 ± 0.013 kPa, respectively (Figure 5i, Figure S4m). The corresponding forces peaked at 103 ± 7 nN in the core, followed by 83 ± 4 nN in the intermediate region, and 66 ± 6 nN even at the periphery (Figure 5j, Figure S4n). Taken together, these data demonstrate that fibroblasts are the primary drivers of escalation of intra‐tumoral compressive stresses, consistent with their role in ECM deposition and contractility.

### 2.6 Temporal Evolution of Mechanical Forces in Tumor–Fibroblast–Macrophage Spheroids

To capture how bulk stresses evolve with spheroid maturation, we focused on tumor–fibroblast–macrophage spheroids, which previously exhibited the strongest stresses and local variations. Spheroids were analyzed on Days 4, 7, and 10, and microparticle deformation was quantified across the core, intermediate, and peripheral regions.

Microparticle sphericity decreased progressively over time in all regions, indicating cumulative deformation (Figure 6a, Movie S3). In the core, sphericity dropped from 0.80 ± 0.022 on Day 4 to 0.73 ± 0.017 on Day 7 and 0.65 ± 0.032 by Day 10. The intermediate region showed a similar decline, from 0.83 ± 0.02 to 0.76 ± 0.024 and 0.70 ± 0.013, while sphericity in the periphery decreased from 0.90 ± 0.021 to 0.84 ± 0.026 and 0.74 ± 0.02 over the same period (Figure 6b). These trends were mirrored by reductions in microparticle volume, with core values decreasing from 1050 ± 40 µm^3^ (Day 4) to 1000 ± 41 µm^3^ (Day 7) and 925 ± 18 µm^3^ (Day 10). Intermediate volumes dropped from 1130 ± 9 µm^3^ to 1067 ± 50 µm^3^ and 975 ± 13 µm^3^, while peripheral volumes contracted from 1225 ± 11 µm^3^ to 1115 ± 25 µm^3^ and 1035 ± 48 µm^3^ (Figure 6c).

**FIGURE 6:**
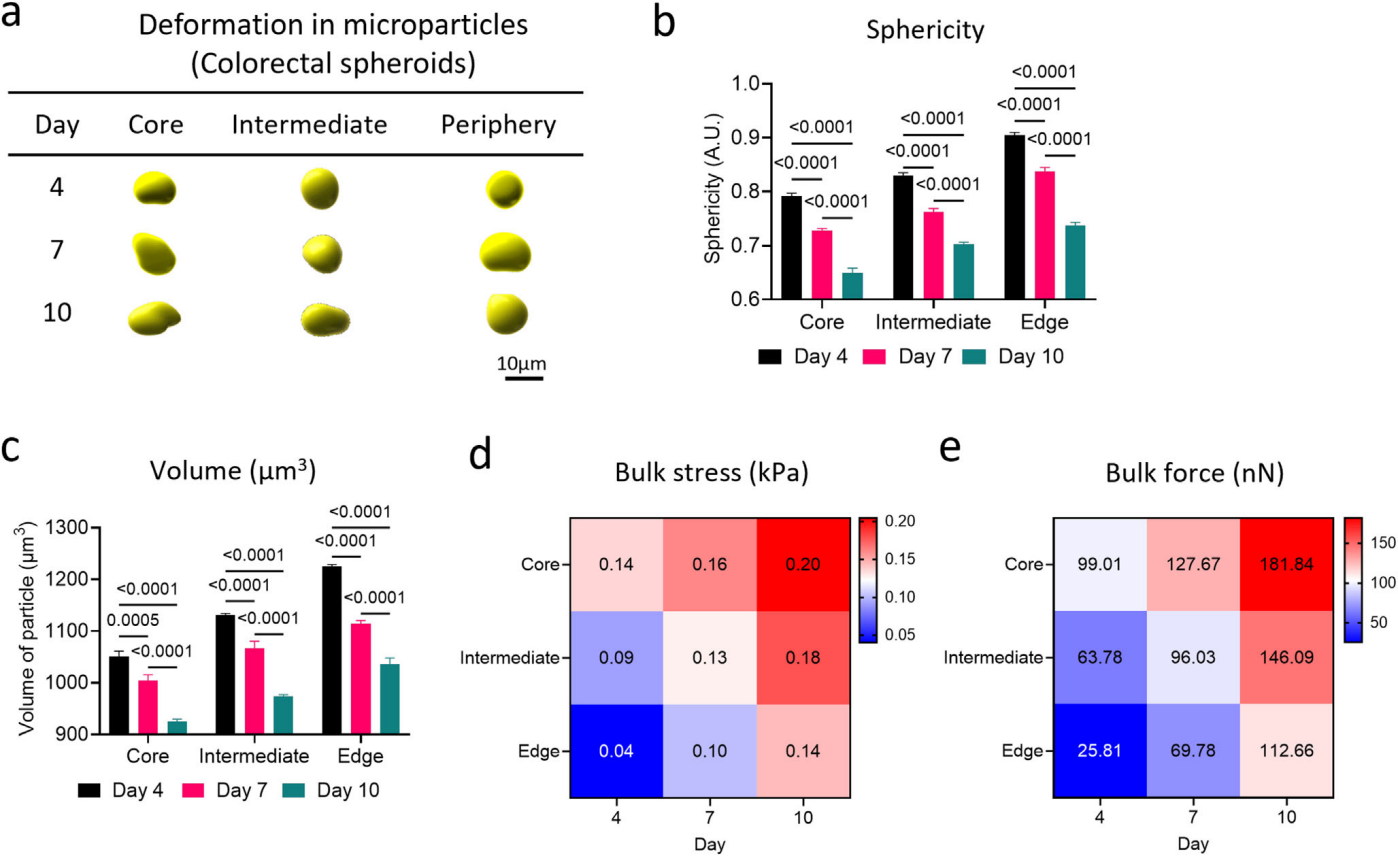
Spatio‐temporal mapping of microparticle deformation reveals fibroblast‐ and macrophage‐driven bulk compressive stresses. (a) Representative images of deformed microparticles in different regions of T + F + M spheroids at different timepoints post spheroid formation showing the change in sphericity due to the bulk compressive forces; (b) Quantification of the sphericity of the microparticles present in different regions of T + F + M spheroids at different timepoints post spheroid formation; (c) Quantification of the volume of the microparticles present in different regions of T + F + M spheroids at different timepoints post spheroid formation; (d) Heatmap showing the quantification of bulk compressive stress in different regions of T + F + M spheroids at different timepoints post spheroid formation as calculated based on the volumetric strain on the microparticles; (e) Heatmap showing the quantification of bulk compressive forces in different regions of T + F + M spheroids at different timepoints post spheroid formation as calculated based on the pressure exerted on the microparticles. Data represented as mean ± standard error of mean (SEM). Statistical analysis for multiple groups comparison was performed with two‐way ANOVA (Tukey).

The increasing compressive deformation translated into progressively higher stresses within the spheroids. In the core, bulk stress rose from 0.013 ± 0.02 kPa on Day 4 to 0.16 ± 0.022 kPa on Day 7 and reached 0.205 ± 0.009 kPa by Day 10 (Figure 6d, Figure S5a). Intermediate bulk stress increased from 0.1 ± 0.005 kPa to 0.13 ± 0.03 kPa and 0.18 ± 0.007 kPa, while peripheral bulk stress rose from 0.1 ± 0.005 kPa to 0.13 ± 0.03 kPa and 0.18 ± 0.007 kPa over the same timeframe (Figure 6d, Figure S5b,c). Interestingly, these calculations indicate that bulk stress becomes more homogeneous in both absolute and relative terms during progressive tumor growth.

Bulk force calculations derived from the stress revealed a striking temporal amplification of mechanical loads. In the core, bulk forces increased from 99 ± 16 nN on Day 4 to 127 ± 18 nN on Day 7 and reached 180 ± 12 nN by Day 10 (Figure 6e, Figure S5d). Intermediate values increased from 63 ± 4 nN to 96 ± 21 nN and 146 ± 7 nN, while peripheral bulk forces escalated especially strongly (> 5‐fold), going from 26 ± 4 to 70 ± 9 nN and ultimately 112 ± 20 nN (Figure 6e, Figure S5e,f).

These findings highlight that the total forces exerted on cell‐sized particles increase substantially over time, particularly in the core where fibroblast–macrophage crosstalk and ECM remodeling are most pronounced.

## 3 Discussion

Solid tumors represent dynamic multicellular ecosystems in which cancer cells interact continuously with stromal and immune cells embedded within a dynamic reorganized ECM [9]. These interactions are known to profoundly influence tumor progression, therapy resistance, and mechanical evolution of the TME [28, 29, 30]. In this study, we established two tri‐cellular 3D spheroid models comprising of murine colorectal cancer cells (CT26), fibroblasts, and macrophages, and human PDAC cells (PANC‐1), primary pancreatic stellate cells (hPSCs), and human monocyte/macrophages [17]. Both these 3D spheroid models were combined with established and optimized deformable hydrogel microparticles to obtain direct, spatial, and temporally resolved measurements of intratumoral bulk stresses. While the deployment of deformable hydrogel beads for internal tissue stress evaluation builds upon foundational work of Dolega et al., its application here to track the macrophage‐fibroblast cross‐talk reveals a previously unquantified dynamic in tumor progression [26]. By systematically comparing tumor‐only, tumor–fibroblast, tumor–macrophage, and tumor–fibroblast–macrophage spheroids, we were able to resolve the distinct and synergistic contributions of fibroblasts and macrophages to tumor growth, gene and protein expression, and mechanical stress generation. Collectively, our results demonstrate that fibroblasts act as primary mechanical drivers of tumor solid stresses through enhanced contractility and ECM deposition, while macrophages amplify these mechanical effects partly by promoting fibroblast activation and shaping an immunosuppressive TME. Overall, this study highlights the fibroblast–macrophage crosstalk as a potent determinant of both biochemical and mechanical tumor evolution.

Fibroblasts played a central role in stimulating tumor cell proliferation which was further enhanced by the inclusion of macrophages (Figure 1). The co‐operative interaction between these two stromal cells established reciprocal signaling network. Such reciprocal proliferation has been described in vivo, where tumor‐associated macrophages secrete mediators such as Tgf‐β, Il‐6, and Pdgf that activate fibroblasts, while fibroblasts in turn can release factors such as Ccl2, Sdf‐1, and Csf‐1 that recruit and polarize macrophages [31, 32]. Our in vitro model recapitulates these mutualistic interactions, demonstrating that fibroblast–macrophage direct contact is sufficient to drive robust expansion even in a simplified 3D environment.

Gene expression analysis provided deeper insights into the molecular basis of these proliferative changes (Figure 2). Tumor cells exhibited changes in epithelial markers and cytoskeletal proteins indicated that fibroblast enhances epithelial plasticity and EMT transition in tumor cells [33, 34]. The synergy in the bulk gene expression analysis was not just an additive effect of the three cell types and their proliferation was analyzed by normalizing the cell‐specific genes with their cell numbers. This analysis further revealed the effect of crosstalk between tumor cells, fibroblasts, and macrophages either via direct contact, or via soluble growth factors. Tgf‐β and Hif1α expression were also markedly elevated, consistent with a stromal driven growth factor signaling, hypoxia, and mechanical constraint. These findings reinforce the view that fibroblasts not only support tumor expansion but also induce EMT in tumor cells [35].

Fibroblast activation markers provided additional evidence for strong stromal reprogramming. Acta2 and Il6 expression patterns reflect the emergence of both myofibroblast and inflammatory CAFs subsets characterized by contractility, cytokine production, and matrix remodeling traits known to be associated with metastasis, immune evasion, and chemoresistance [36, 37]. The synergy between fibroblasts and macrophages promotes macrophage‐derived Il‐1β, Il‐10, and Tgf‐β known to induce CAF activation, whereas CAF‐secreted Il‐6, Sdf‐1, and Opn can skew macrophages toward an M2‐like immunosuppressive phenotype. The pronounced upregulation of Arg1, a canonical M2 marker, in the tri‐culture spheroids confirms the strength of this skewing and suggests that macrophages adopt a pro‐tumoral phenotype under the combined influence of fibroblasts and tumor cells [32, 36, 37, 38].

Perhaps the most striking changes were observed in ECM‐related gene expression. Collagen, fibronectin, and integrin transcripts were highly elevated, suggesting a strong mechanobiological remodeling as a function of fibroblast‐macrophage crosstalk. ECM deposition and integrin signaling are central components of mechanotransduction, linking biochemical changes in the microenvironment to physical changes in tissue stiffness and force transmission [3, 39, 40]. The synergistic increase in Arg1 and Tgf‐β suggests a feedback loop where M2‐polarized macrophages provide the biochemical cues necessary for robust CAF activation. This paracrine signaling results in the elevated traction forces and ECM remodeling that we quantified via microparticle mapping. Targeting this axis, for instance through Tgf‐β inhibition or macrophage re‐polarization, represents a viable strategy to ‘normalize’ the tumor mechanics and potentially improve therapeutic efficacy. The observed stromal behavior supports the view that fibroblasts generate substantial mechanical forces, while the macrophages amplify the mechanical forces reshaping tissue architecture.

The integration of deformable microparticles provided a direct method to quantify the mechanical changes. This approach enabled spatial estimation of both isotropic compressive stresses and anisotropic shape deformations (indirectly measured using sphericity) [22, 24, 41]. The mechanical dynamics align with enhanced contractility (Acta2 or αSma), ECM production (Col1α1, Fn1), and Tgf‐β signaling. Contractile fibroblasts apply traction forces on the ECM and on neighboring cells, generating local stresses that accumulate within the confined 3D spheroids [3, 40, 42]. As the matrix becomes denser, its resistance to deformation increases, amplifying mechanical feedback loops that further enhance fibroblast activation [43]. Macrophages appear to potentiate this process by promoting CAF activation and expanding fibroblast numbers. The high nuclear localization of Yap demonstrates that tumor cells themselves respond mechanically to these stromal‐driven forces through the YAP signaling pathway [44]. Because Yap is a central mechanotransducer regulating proliferation, survival, and migration, its activation may help explain the enhanced tumor expansion observed in tri‐culture spheroids [3, 45, 46]. While global tumor stiffness is often measured via macroscopic rheology, our microparticle‐based approach provides a high‐resolution map of the internal solid stresses which is a primary driver of mechanotransduction signaling like YAP.

It is important to contextualize the magnitude of our measured solid stresses (∼0.2 kPa in tri‐culture cores) relative to prior literature. Previous studies, such as Dolega et al., reported internal stress values in the range of 0.5–1 kPa. However, those higher values reflect models subjected to external mechanical loading or osmotic compression using dextran [26]. In contrast, our approach quantifies endogenous solid stress generated purely by internal cellular activity such as active contractility, matrix deposition, and volumetric growth in an unconstrained 3D environment. Furthermore, utilizing soft hydrogel microparticles (*E* = 0.3 kPa) provides high physical sensitivity, enabling the detection of these subtle, internally generated growth and traction forces without applying external pressure artifacts.

While cell proliferation contributes to physical crowding within 3D spheroids, our comparisons confirm that active fibroblast contractility and matrix remodeling are the principal factors of increased force. In T + M spheroids, cell numbers increased relative to baseline, yet bulk stresses remained low and indistinguishable from tumor monocultures (∼0.06 kPa). Elevated compressive stress was observed exclusively in fibroblast‐containing spheroids (T + F and T + F + M), directly matching the dramatic upregulation of Acta2 and Col1α1. Even when accounting for total cell counts at Day 7, the calculated stress was substantially higher in tri‐cultures, proving that phenotypic myofibroblast contractility and matrix contraction amplified by macrophage crosstalk drive the mechanical landscape rather than passive volumetric growth alone.

Spatial immunofluorescence analysis provides a physical explanation for the observed stress gradients. In tri‐culture spheroids, activated fibroblasts and M2‐like macrophages are heavily enriched within the core and intermediate zones, directly coinciding with the spatial peak in compressive stress (0.18–0.23 kPa) and nuclear YAP localization. Furthermore, the marked loss of microparticle sphericity (dropping to 0.72 in colorectal and 0.55 in PDAC cores) serves as an indirect quantitative readout of anisotropic (directional) stress. On top of experiencing isotropic pressure, the embedded particles are subjected to asymmetric squeezing, reflecting directional traction forces exerted by contractile fibroblast networks and ECM in the core.

Temporal analysis of mechanical evolution revealed that compressive stress and force increased progressively with spheroid maturation [47]. Interestingly, the spatial gradients of stress became more homogeneous over time, likely reflecting ECM remodeling and increasing mechanical connectivity among regions [48]. Early in spheroid development, locally contractile fibroblast populations may generate uneven force fields, but as ECM accumulates and stiffens, the entire spheroid becomes mechanically integrated. This transition from spatially heterogeneous to more uniformly distributed stress is reminiscent of in vivo tumor progression, where desmoplastic remodeling leads to globally elevated solid stress that compresses blood vessels, restricts perfusion, and contributes to hypoxia [49, 50, 51]. The rising Hif1α expression we observed supports this connection.

Together, these findings highlight that tumor mechanics emerge not simply from tumor cell proliferation but from cooperative interactions between tumor cells, fibroblasts, and macrophages that jointly reshape both biochemical signaling and the physical microenvironment [40]. Importantly, fibroblasts act as the principal generators of mechanical stress through ECM secretion and contractility, while macrophages amplify this effect by enhancing fibroblast activation and adopting an immunosuppressive phenotype [52, 53, 54]. This synergistic remodeling of tumor mechanics has significant implications. Elevated compressive stresses have been shown to inhibit drug penetration, promote cancer stemness, facilitate invasion, and limit cytotoxic immune cell penetration [55, 56, 57]. Thus, the mechanical consequences of fibroblast–macrophage interactions likely extend beyond the growth effects observed here, influencing broader aspects of tumor biology.

This study also validates deformable microparticles as a robust tool for quantifying mechanical stresses within complex multicellular assemblies. Unlike traction force microscopy, which relies on cell–substrate interactions, or bulk rheology, which lacks spatial resolution, microparticle‐based sensing provides cell‐scale measurements directly within the 3D context. Our data show that microparticles can detect subtle differences in mechanical loading between conditions and track stress evolution over time. Integrating such mechanical readouts with gene and protein expression data strengthens mechanobiological interpretations and opens new opportunities to study how mechanical feedback regulates cell behavior.

There are certain limitations to consider. First, although fibroblasts and macrophages are two of the most important stromal components, other cells present in the TME such as endothelial cells, pericytes, neutrophils, and adaptive immune cells also contribute to mechanical remodeling and could dramatically influence the mechanical landscape in vivo. Incorporating additional cell types or vascular elements could further refine the model. Second, hydrogel microparticles measure local compressive stresses but only indirectly capture shear stresses or tensile forces, which also play important roles in tumor mechanics. Third, although we observed strong correlations between gene expression and mechanical changes, the question whether mechanical stress enhances gene expression or vice versa remains to be fully studied. Moreover, the aforementioned studies using inhibitors of contractility (e.g., Rock inhibitors, TGF‐β inhibitor) or macrophage polarization (CSF‐1R inhibitor, or bisphosphonates‐ to deplete macrophages) could help dissect these interactions.

## 4 Conclusion

This study provides compelling evidence that fibroblast–macrophage interactions are key amplifiers of mechanical stress within tumors and that their synergy induces the tumor growth and mechanotransduction. By integrating multicellular spheroid models with direct mechanical measurements, we could capture a more resolved understanding of how biochemical and biophysical change during tumor progression. This combined approach is well suited for future studies to improve the understanding of the mechanical part of the TME and also to design strategies to improve the efficacy of cancer therapeutics. Ultimately, our findings highlight the importance of considering tumor mechanics as a crucial hallmark that guides the complex cellular interactions within the TME.

## 5 Materials and Methods

### 5.1 Cell Culture

Mouse CT26 colorectal carcinoma cells were a kind gift from Dr. Hawinkels, Leiden University Medical Centre, The Netherlands. 3T3 fibroblasts (#ATCC CRL‐1658), murine macrophages RAW264.7 (#ATCC TIB‐71), human PANC‐1 PDAC cells (#ATCC CRL‐1469), and THP‐1 cells (#ATCC TIB‐202) were obtained from the American Type Culture Collection (ATCC, Manassas, VA, USA). Human pancreatic stellate cells (hPSCs) were obtained from ScienCell (Carlsbad, USA).

CT26, RAW264.7, and THP‐1 cells were cultured in RPMI medium (Lonza Basel Switzerland) supplemented with 10% fetal bovine serum (FBS, Sigma‐Aldrich, St. Louis, MO, USA), 100 U/mL penicillin (Thermofisher Scientific, Waltham, MA, USA), 100 µg/mL streptomycin (Thermofisher Scientific) and 2 mM l‐glutamine (Thermofisher Scientific). NIH3T3 cells and PANC‐1 cells were cultured in Dulbecco's Modified Eagle Medium (DMEM, Lonza, Basel, Switzerland) supplemented with 10% fetal bovine serum (FBS, Sigma‐Aldrich, St. Louis, MO, USA), 100 U/mL penicillin (Thermofisher Scientific, Waltham, MA, USA), 100 µg/mL streptomycin (Thermofisher Scientific) and 2 mM l‐glutamine (Thermofisher Scientific). hPSCs were cultured in stellate cell medium (ScienCell, Carlsbad, USA) supplemented with 2% FBS, 1% stellate cell growth supplement (SteCGS), and 1% penicillin/streptomycin solution. All cell types were cultured in a humidified atmosphere of 5% CO_2_ at 37°C. Cultures were split at 80% confluence. RAW264.7 cells were kept between 10 and 20 passages. hPSCs were cultured from passage 4 to 10 and THP‐1 cells were cultured between passage 10 and 25.

### 5.2 Spheroid Formation

Tumor spheroids were generated using CT26 colon cancer cells either alone or in co‐culture with 3T3 fibroblasts and/or RAW267.4 murine macrophages for colorectal spheroids and using PANC‐1 PDAC tumor cells either alone or in co‐culture with human pancreatic stellate cells (hPSCs) and/or THP‐1 human monocytes. In brief, spheroids were generated by culturing CT26 colon cancer cells or PANC‐1 PDAC cancer cells (6000 cells/well) in a round‐bottom 96 well plate that has been coated overnight with 1% (w/v) Pluronic F127 (Sigma‐Aldrich) at 37°C, washed with sterile MiliQ and air‐dried. Multicellular spheroids were assembled using the same process, with total cell numbers being 6000, in the following ratios: T + F (1:5; 1000 tumor cells + 5000 fibroblasts), T + M (1:0.05; 5714 tumor cells + 286 macrophages), and T + F + M (1:4.95:0.05; 1000 tumor cells + 4950 fibroblasts + 50 macrophages). All spheroids were cultured in 1:1 RPMI: DMEM supplemented with 10% FBS and 1% penicillin‐streptomycin and maintained at 37°C with 5% CO_2_. Prior culture of spheroids containing murine macrophages, RAW264.7 cells were treated with mytomicin‐C (10 µg/mL, 2 h). Growth and morphology were monitored by brightfield microscopy on Days 3, 5, and 7.

In order to understand the stiffness inside the spheroids, 20 000 microparticles per spheroid were mixed with the cell suspension before seeding them in a 96 well plate. Spheroids self‐assembled with the microparticles incorporated inside. These cultures were allowed to form for 3 days of undisturbed culture at 37°C.

### 5.3 Microparticle Synthesis

Deformable acrylamide microparticles were synthesized using a membrane‐emulsification approach as previously described [24]. Acrylamide mixtures containing 100 mg/mL acrylic components, 150 mM NaOH, 0.3% (v/v) tetramethylethylenediamine were prepared in 150 mM MOPS buffer (pH 7.4, made from MOPS sodium salt). Acrylic acid mass fraction 10%, whereas crosslinker mass fraction was 0.32%. The gel mixture was degassed for 15 min and kept under nitrogen atmosphere until emulsification. The 1.9 µm tubular hydrophobic Shirasu porous glass (SPG) membranes were sonicated in n‐heptane under vacuum, mounted on an internal pressure micro kit extruder (SPG Technology Co.) and immersed into an oil phase (∼125 mL) consisting of hexanes and 3% (v/v) Span 80 (Sigma‐Aldrich, S6760). 10 mL of gel mixture was extruded into the oil phase under nitrogen pressure of approximately 7 kPa, while the oil phase was stirred continuously at 300 rpm. After extrusion, ∼225 mg 2,2’‐Azobisisobutyronitrile (AIBN) (1.5 mg/mL final concentration) was added to induce polymerization of microparticles at 60°C. After 3 h, the emulsion (now dispersion) was cooled down, and polymerized microparticles were subsequently washed (5× in hexanes, 1× in ethanol), dried under nitrogen flow for approximately 30 min, and resuspended in PBS (137 mM NaCl, 2.7 mM KCl, 8.0 mM Na_2_HPO_4_, 1.47 mM KH_2_PO_4_, pH 7.4) and stored at 4°C. The final particles diameter was ∼13 µm. The Young's modulus (*E*) of particles of this composition was previously determined to be 0.30 kPa and using osmotic compression it was also determined that acrylamide microparticles have a Poisson's ratio (ν) of ∼0.43 [24]. The bulk modulus (*K*) was then determined using the equation *K* = *E*/[3(1 − 2*ν*)], yielding a value of 0.71 kPa.

### 5.4 Microparticle Functionalization

DAAM‐particles were functionalized as previously described [24]. In brief, DAAM‐particles were diluted to 5% (v/v) concentration and washed twice in activation buffer (100 mM MES, 200 mM NaCl, pH 6.0). They were then incubated, under gentle mixing, for 15 min in activation buffer supplemented with 40 mg/mL 1‐ethyl‐3‐(3‐dimethylaminopropyl) carbodiimide, 20 mg/mL N‐hydroxysuccinimide (NHS) and 0.1% (v/v) Tween 20. They were then spun down (10 000 × *g*, 1 min) and washed, each time mixing vigorously, 4× in PBS, pH 8 (adjusted with NaOH) with 0.1% Tween 20. Particles were then resuspended in 10 mg/mL BSA (Sigma, A3059) in PBS, pH 8 and incubated while rocking for 1 h. Then Tetramethylrhodamine Cadaverine (Thermo Fisher Scientific, A1318) with final concentration 0.2 mM. After 30 min, 100 mM Tris and 100 mM ethanolamine (pH 9) were added to block unreacted NHS groups. DAAM‐particles were then spun down (16 000 × *g*, 2 min) and washed 4× in PBS, pH 7.4 with 0.1% Tween 20.

### 5.5 Flow Cytometry

Formed spheroids were pooled together in a falcon tube with 20 spheroids per condition to have enough cells to be gated. Spheroids were dissociated into single‐cell suspensions using 0.5 mg/mL collagenase and 0.5 mg/mL dispase in RPMI medium. Once spheroids were completely dissociated, the cell suspension was processed for flow cytometry. To count the total number of cells, the cells were only stained with Hoechst for 10 min and then used in the flow cytometer. Cell‐type‐specific populations like tumor cells, fibroblasts, and macrophages were quantified, using cell specific antibodies mentioned in the Table 1. The count of antibody stained cells was used to determine the proliferation rates of these cell types.

**TABLE 1:** List of antibodies used for flow cytometry.

Primary antibody	Source	Host	Dilution
Ck19	Novus biotech	Rabbit	1:200
αSMA	Sigma Aldrich	Mouse	1:500
F4/80	Thermo Fisher	Rat	1:200
488 Alexa Fluor anti mouse	Thermo Fisher	Donkey	1:100
488 Alexa Fluor anti rat	Thermo Fisher	Goat	1:100
488 Alexa Fluor anti rabbit	Thermo Fisher	Donkey	1:100

### 5.6 Gene Expression Analysis

Spheroids were first dissociated into a single cell suspension as described in flow cytometry. The dissociated spheroids were centrifuged to achieve a cell pellet before RNA isolation. The total RNA was isolated using the GenElute Mammalian Total RNA Miniprep Kit (Sigma‐Aldrich) and the RNA amount was measured by a Nanodrop ND‐1000 Spectrophotometer (Thermofisher Scientific). Next, cDNA was synthesized with iScript cDNA Synthesis Kit (BioRad, Veenendaal, the Netherlands). For each PCR reaction 10 ng cDNA was used. All real‐time PCR primers (Table 2) were purchased from Sigma‐Aldrich. Quantitative real time PCR was performed with the 2× Sensimix SYBR and Flurescein Kit (Bioline GmBH, Luckenwalde, Germany) using a BioRad CFX384 real‐time PCR detection system (BioRad). Relative expression was normalized to housekeeping gene GAPDH.

**TABLE 2:** List of primers.

Gene	Forward primer	Reverse primer
E‐cadherin (Cdh1)	AACCCAAGCACGTATCAGGG	GAGTGTTGGGGGCATCATCA
Vimentin (Vim)	TCCAGAGAGAGGAAGCCGAA	AAGGTCAAGACGTGCCAGAG
Tgf‐β	GGACTCTCCACCTGCAAGAC	CTGGCGAGCCTTAGTTTGGA
Hif1α	TCAAGTCAGCAACGTGGAAG	TATCGAGGCTGTGTCGACTG
Acta2 (αSMA)	ACTACTGCCGAGCGTGAGAT	CCAATGAAAGATGGCTGGAA
Interleukin 6 (Il6)	TGATGCTGGTGACAACCACGGC	TAAGCCTCCGACTTGTGAAGTGGTA
Collagen1α1	TGACTGGAAGAGCGGAGAGT	ATCCATCGGTCATGCTCTCT
Fibronectin (Fn)	ATGAGAAGCCTGGATCCCCT	GGAAGGGTAACCAGTTGGGG
F4/80	TGCATCTAGCAATGGACAGC	GCCTTCTGGATCCATTTGAA
Arginase1	GTGAAGAACCCACGGTCTGT	CTGGTTGTCAGGGGAGTGTT
Mhc‐II	TCCAGATGCCAACGTGGCCC	TGCGGAAGAGGTGATCGTCCC
Il1β	GCCAAGACAGGTCGCTCAGGG	CCCCCACACGTTGACAGCTAGG
Tnfα	AGGCTGCCCCGACTACGTGC	CAGCGCTGAGTTGGTCCCCC
Ck19	TCTGAGCTACCAGCGAGACT	ACAAGTCGAGGGAGGGGTTA
Itgav	CACGTCCTCCAGGATGTTTC	AAACTCAATGGGCTGGCACC
Itgβ1	GCAAATGCCAAATCTTGCGG	TGGCTGGCAACCCTTCTTTT
Mmp2	TTTCTATGGCTGCCCCAAGG	GTCAAGGTCACCTGTCTGGG
Mmp3	GTTCTGGGCTATACGAGGGC	TTCTTCACGGTTGCAGGGAG
Mmp9	CGTCGTGATCCCCACTTACT	AACACACAGGGTTTGCCTTC
Mmp10	TGCTGCCTATGAGGCTCACAAC	GGAGGAAAACCGAGAGTGTGGA
Mmp13	CCAGAACTTCCCAACCATGT	GTCTTCCCCGTGTTCTCAAA
Timp1	ATCAGTGCCTGCAGCTTCTT	TGACGGCTCTGGTAGTCCTC

For normalized gene expression to the specific cell‐type, we divided by the gene expression by the total number of cells of that specific type which were counted using flow cytometric analysis. Then the gene expression of one of the spheroid was set at 1 to get the fold change of other spheroids.

### 5.7 Protein Expression

Spheroids were formed as previously described before being washed with DPBS, fixed with 4% formaldehyde (Sigma‐Aldrich), washed again with DPBS and being embedded into Cryomatrix (Thermofisher Scientific) before being snap‐frozen using isopentane (Sigma‐Aldrich). Embedded and frozen spheroids were cut into 6 µm thick cryosections, air‐dried, and fixed with acetone for 10 min at room temperature. A circle was drawn around the tissues using a hydrophobic PAP pen (Sigma‐Aldrich) before being rehydrated in PBS. The rehydrated sections were incubated overnight in primary antibodies against various markers, listed in Table 3, overnight at 4°C. Next, the sections were washed with PBS, before being incubated with fluorescent secondary antibodies (Table 3), washed again in PBS and mounted using Fluoroshield with DAPI and imaged using Nanozoomer‐RS.

**TABLE 3:** List of antibodies used for immunofluorescence staining.

Primary antibody	Source	Host	Dilution
Ck19	Novus biotech	Rabbit	1:200
αSMA	Sigma Aldrich	Mouse	1:500
F4/80	Thermo Fisher	Rat	1:200
Collagen	Southern Biotech	Goat	1:200
Arginase1	Santa Cruz Biotech	Rabbit	1:200
YAP	Santa Cruz Biotech	Rabbit	1:200
488 Alexa Fluor anti mouse	Thermo Fisher	Donkey	1:100
488 Alexa Fluor anti rat	Thermo Fisher	Goat	1:100
488 Alexa Fluor anti rabbit	Thermo Fisher	Donkey	1:100
488 Alexa Fluor anti goat	Thermo Fisher	Donkey	1:100

### 5.8 Confocal Imaging and 3D Reconstruction Using IMARIS

Spheroids containing microparticles were formed as described earlier. For spatial mapping, tumor spheroids or multicellular spheroids were collected 7 days after their initial seeding. They were washed twice with DPBS. Samples were directly counterstained with DAPI before visualization under the confocal microscope. Spheroids were embedded on glass slides with a cover slip to minimize movement during imaging. Z‐stacks were acquired using a confocal laser scanning Zeiss LSM series microscope. Excitation was performed using 405 nm (DAPI) and 561 nm (Alexa Fluor 568) lasers, and emission signals were collected using sequential scanning mode to minimize spectral overlap. Z‐stacks were collected at 1 µm intervals across the full spheroid thickness, ensuring Nyquist sampling for subsequent 3D reconstruction.

Similarly, for temporal mapping of the microparticles, spheroids were collected at different timepoints (Days 4, 7, and 10). Same procedure was followed as described for spatial mapping of the microparticles.

For microparticle deformation analysis, spheroids containing fluorescently labeled microparticles were imaged under identical acquisition settings. Microparticles were identified based on fluorescence intensity, segmented using IMARIS software, and reconstructed in 3D using the surface rendering module. Particle sphericity, volume, surface area and position of the particles in the 3D space were calculated from reconstructed surfaces, with sub‐voxel resolution achieved by applying Gaussian smoothing prior to analysis. All the microparticles per spheroid type and region (core, intermediate, periphery) were analyzed to ensure statistical robustness. According to the position of the microparticles, they were sub‐divided into different regions by shifting the origin to the center of the spheroid.

All imaging parameters (laser power, gain, offset, and pinhole size) were kept constant across experimental groups to allow direct comparison of fluorescence intensity and particle deformation.

### 5.9 Calculation of Pressure and Force From Microparticle Deformation

To quantify the bulk compressive mechanical stresses within the spheroids, we calculated the pressure and force acting on embedded microparticles based on their volumetric deformation. The undeformed volume of the highly monodisperse (coefficient of variation < 0.1) microparticles (*V*_0_) was measured as 1300 µm^3^. After spheroid embedding and culture, the deformed particle volumes (*V*) and sphericities (*ϕ*) were obtained by 3D image reconstruction. The volumetric strain (*ε*) was defined as: *ε* = ((V_0_ − V))/*V*_0._

Pressure was then calculated using the linear equation as: Pressure (*P*) = Bulk modulus (*K*) * Volumetric strain (*ε*). Knowing the pressure, the force on the microparticles was calculated using the equation as: Force = Pressure (*P*) * Total Surface Area (*A*) of the microparticles after deformation. The microparticle area obtained from IMARIS was used to calculate the force.

From this the force applied on the microparticle was calculated using: F = P*A.

For each experimental group, values of pressure and force were calculated for individual particles and then averaged across particles within the spheroid core, intermediate region, and periphery. Results are expressed as mean ± standard deviation. This approach assumes that the microparticles behave as linearly elastic, isotropic spheres.

### 5.10 Statistical Analysis

All graphs were made using GraphPad Prism (Version 10.6.0, GraphPad Software Inc., San Diego, CA, USA) based on calculations using Microsoft Excel. All values are expressed as mean ± standard error of the mean (SEM). Statistical significance of the results was performed by Ordinary one‐way ANOVA using Tukey's multiple comparisons test to compare between different groups in a particular graph. For multiple comparison between different groups, 2way ANOVA with Tukey's multiple comparisons were used. Significant difference was determined for a *p* value of *p < 0.05, **p < 0.01, and ***p < 0.001 respectively.

## Author Contributions

**Kunal P. Pednekar**: conceptualization, methodology, software, data curation, investigation, validation, formal analysis, writing – original draft, writing – review and editing. **Daan Vorselen**: conceptualization, supervision, writing – review and editing, methodology. Jai Prakash: conceptualization, methodology, supervision, funding acquisition, project administration, writing – review and editing.

## Funding

This study was supported by funding from the Phospholipid Research Center (JPR‐2020‐088/3‐1 to Jai Prakash) and European Research Council (ERC Advanced Grant, project number 101142157 to Jai Prakash).

## Conflicts of Interest

The authors declare no conflicts of interest.

## Supporting information




**Supporting File 1**: adhm71844‐sup‐0001‐SuppMat.docx.

**Supporting File 2**: adhm71844‐sup‐0002‐MovieS1.mp4.

**Supporting File 3**: adhm71844‐sup‐0003‐MovieS2.mp4.

**Supporting File 4**: adhm71844‐sup‐0004‐MovieS3.mp4.

## Data Availability

The data that support the findings of this study are available from the corresponding author upon reasonable request.
