# Supplementary material for: Macrophage–Fibroblast Crosstalk Governs Tumor Mechanics, as Revealed by Spatiotemporal Force Mapping in 3D Spheroids
Source: Adv Healthc Mater. 2026 Oct 16;15(42):e71844. doi: 10.1002/adhm.71844 (PMC13768204; doi:10.1002/adhm.71844)
Supplement: Supplementary file 1 [file ADHM-15-0-s002.docx]

**Macrophage–Fibroblast Crosstalk Governs Tumor Mechanics, as Revealed by Spatiotemporal Force Mapping in 3D Spheroids**

Kunal P. Pednekar, Daan Vorselen, Jai Prakash^*^

K. P. Pednekar, J. Prakash

1) Department of Medical Biosciences, Radboud University Medical Centre, Geert Grooteplein Zuid 10, 6525 GA, Nijmegen, The Netherlands.

2) Department of Bioengineering Technologies, University of Twente, Drienerlolaan 5, 7500AE, Enschede, The Netherlands.

E-mail: [kunal.pednekar@radboudumc.nl](mailto:kunal.pednekar@radboudumc.nl), [jai.prakash@radboudumc.nl](mailto:jai.prakash@radboudumc.nl)

D. Vorselen

3) Department. of Cell Biology & Immunology (CBI), Wageningen University & Research, De Elst 1, 6708WD, Wageningen, The Netherlands

E-mail: [daan.vorselen@wur.nl](mailto:daan.vorselen@wur.nl)

***Corresponding author**

**Funding:** This study was supported by the funding from Phospholipid Research Center (JPR-2020-088/3-1 to J.P.) and European Research Council (ERC Advanced Grant, project number 101142157 to J.P.).

**Keywords:** tumor stroma, macrophages, fibroblasts, extracellular matrix, force mapping, tumor spheroids, 3D cancer models

**Supplementary Figure 1: Spheroid growth in PDAC mono- and co-culture spheroids. (a)** Representative brightfield microscopic images of PDAC spheroids formed using the tumor cells alone, or either co-cultured with fibroblasts or macrophages, or both fibroblast and macrophages, (b) Line graph showing the growth curve of PDAC spheroids as measured from the brightfield microscopic images. Each point is an average of at least 10 spheroids (technical replicate) and each line is a biological replicate of the spheroid growth, (c) Representative fluorescent images of live and dead staining and the stacked bar graph showing the quantification of live and dead cell percentage. Data represented as mean ± standard error of mean (SEM).

**Supplementary Figure 2: Gene expression of different types of spheroids.** Bar graph showing the gene expression of Itgαv, Itgβ, Il1β, Tnfα, Mmp2, Mmp3, Mmp9, Mmp10, Mmp13, and Timp1. Data represented as mean ± standard error of mean (SEM). Statistical analysis for multiple groups was performed with one way ANOVA (Tukey).

**Supplementary Figure 3:** (a) Schematic representation of the distribution of microparticles in different regions of the spheroids and the interpretation of concentric regions from at least 50 spheroids depending on the change in phase contrasts over the regions, (b) Bar graph showing the DAPI per unit area (spheroid compactness) for different types of colorectal spheroids. Data represented as mean ± standard error of mean (SEM). Statistical analysis for multiple groups was performed with one way ANOVA (Tukey).

**Supplementary Figure 4: Spatial bulk stress and force quantification.** (a-h) Bar graphs showing the quantification of bulk compressive stress (kPa, a, c, e, g) and bulk compressive force (nN, b, d, f, h) in different regions of different types of colorectal spheroids tumor only (a, b), T+F (c, d), T+M (e, f), and T+F+M (g, h), (i-n) Bar graphs showing the quantification of bulk compressive stress (kPa, i, k, m) and bulk compressive force (nN, j, l, n) in different regions of different types of PDAC spheroids tumor only (i, j), T+F (k, l), and T+F+M (m, n). Statistical analysis for multiple groups was performed with one way ANOVA (Tukey).

**Supplementary Figure 5: Spatio-temporal bulk stress and force quantification.** (a-f) Bar graphs showing the quantification of bulk compressive stress (kPa, a, b, c) and bulk compressive force (nN, d, e, f) in different regions- core (a, d), intermediate (b, e), and edge (c, f) of T+F+M spheroids at different time points. Statistical analysis for multiple groups was performed with one-way ANOVA (Tukey).

**Movie 1**. **Video showing spatial mapping of microparticles incorporated in colorectal spheroids.** A panel of videos showing spatial mapping of deformed microparticles within different spheroids in relation to data presented in figure 5a.

**Movie 2**. **Video showing spatial mapping of microparticles incorporated in PDAC spheroids.** A panel of videos showing spatial mapping of deformed microparticles within different spheroids in relation to data presented in figure 5f.

**Movie 3**. **Video showing temporal mapping of microparticles incorporated in colorectal spheroids.** A panel of videos showing temporal mapping of deformed microparticles at different timepoints in tri-cultured spheroids in relation to data presented in figure 6a.
